# 

**Published:** 1897-04-03

**Authors:** 


					rna n\jorli'AL. ABbU.LU 1 HL. x FXTKE^ tnereiore cc.di. armr, .skp, i?yy.
OADBURY'S U? w, CADBURY'S
COOOA ;lfe^ COOOA
^^hWj?tpu"tyatpresent Jb ^ ^ NO ALKALIES USED
\}uiU/r>/
<s\mWXCJ^
y'9r
WouuITnofi men HOSPI TAL "
No. 549/ Yol. XXn;LSfiS5Ua Saturday,
April 3rd, 1897.
[ Tee p.Verm J PRICE TWOPENCE.

				

